# Mathematical surface function-based design and 3D printing of airway stents

**DOI:** 10.1186/s41205-022-00154-8

**Published:** 2022-08-06

**Authors:** Bengi Yilmaz, Bilge Yilmaz Kara

**Affiliations:** 1 Department of Biomaterials, University of Health Sciences Turkey, 34668 Istanbul, Turkey; 2 Experimental Medicine Research and Application Center, University of Health Sciences Turkey, 34662 Istanbul, Turkey; 3 Department of Pulmonary Medicine, Recep Tayyip Erdoğan University School of Medicine, 53020 Rize, Turkey

**Keywords:** Airway stents, 3D printing, Design, Vat polymerization, Stereolithography, Additive manufacturing

## Abstract

**Background:**

Three-dimensional (3D) printing is a method applied to build a 3D object of any shape from a digital model, and it provides crucial advantages especially for transferring patient-specific designs to clinical settings. The main purpose of this study is to introduce the newly designed complex airway stent models that are created through mathematical functions and manufactured with 3D printing for implementation in real life.

**Methods:**

A mathematical modeling software (MathMod) was used to design five different airway stents. The highly porous structures with designated scales were fabricated by utilizing a stereolithography-based 3D printing technology. The fine details in the microstructure of 3D printed parts were observed by a scanning electron microscope (SEM). The mechanical properties of airway stents with various designs and porosity were compared by compression test.

**Results:**

The outputs of the mathematical modeling software were successfully converted into 3D printable files and airway stents with a porosity of more than 85% were 3D printed. SEM images revealed the layered topography of high-resolution 3D printed parts. Compression tests have shown that the mathematical function-based design offers the opportunity to adjust the mechanical strength of airway stents without changing the material or manufacturing method.

**Conclusions:**

A novel approach, which includes mathematical function-based design and 3D printing technology, is proposed in this study for the fabrication of airway stents as a promising tool for future treatments of central airway pathologies.

**Supplementary Information:**

The online version contains supplementary material available at 10.1186/s41205-022-00154-8.

## Background

Airway stents are hollow tubular endobronchial prostheses composed of a variety of biodegradable or non-biodegradable materials that support and maintain the patency of the airways. Indications for airway stenting can occur in both malignant and benign processes. The conditions related to malignancy, such as endobronchial tumor with residual obstruction after multimodality thermal therapy, malignant airway obstruction from extrinsic compression, loss of cartilage support from tumor destruction and malignant tracheoesophageal fistula, and the benign diseases, such as complex benign stricture or stenosis > 4 cm in length, benign stricture or stenosis in an inoperable patient, post-transplant airway stenosis and benign tracheoesophageal fistula, are the possible indications of airway stenting [1]. The use of an airway stent might be necessary to prevent a tumor from spreading throughout the airway, to aid the healing of airway fistulas, or to prevent the airway wall from collapsing. An "ideal" stent should have the mechanical strength to withstand external pressure forces to maintain the lumen patency and it should also be flexible enough to conform to the shape of the airway lumen. In addition, it should be biocompatible, non-migratory and should act as a barrier against the ingrowth of the tumor if present. In addition, stents are foreign bodies that can cause complications such as bacterial colonization and invasive infections which may sometimes require immediate intervention [2]. The most common complications of airway stents can be listed as migration of the stent, formation of granulation tissue around the stent, problems with mucociliary clearance, low patient tolerance, problems with placement and removal, and breakage of the stent [3]. It is hoped that in the future 3D printing technology can help researchers make significant advances in the search for an ideal airway stent design and manufacture [4].

Airway stents are produced in certain forms such as straight, branched Y and T tube shapes. Most manufacturers can only offer customization in sizes and diameters and each different stent design has its own technical limitations. For example, the most widely used silicone stent—the DUMON stent with a studded cylindrical tube shape—is more likely to result in accumulation and blockage of mucous when used in the management of benign airway stenosis, and there is no effective way to avoid this [5]. Therefore, it must be removed when necessary. Also, as commercially available Y-stents are designed to be placed in the trachea, they may be too large to be placed in the right main stem bronchus in some patients [6].

There is hardly any field in medicine that does not take advantage of the new opportunities in biomedical technology. While initially only anatomical models and surgical planning parts created from patients’ medical scanning images were 3D printed, now many final products are made with the same techniques [7]. A milestone in pulmonary medicine was the creation of a biodegradable 3D printed airway splint that was implanted in a child with bronchomalacia at University of Michigan in 2013 [8]. One year later, a multidisciplinary effort was made to produce a computer-designed bioabsorbable tracheobronchial splint and it was tested in a severe tracheobronchomalacia model in pigs and found to prolong survival [9]. A very important breakthrough came with a study by a large research team from ETH Zurich in 2021 [10]. The multidisciplinary team reported the vat (tank) polymerization 3D printing of customized and bioresorbable airway stents that exhibit tunable elastomeric properties and suitable biodegradability intending to provide a better solution for the central airway obstruction over standard-of-care silicone tube airway stents that are prone to migration. These attempts are still very new and currently limited to just a few studies. Airway stenting technology is lagging behind other specialties in benefiting from advances in biomaterials and tissue engineering technologies however there are new developments revealing the possibility of similar improvements in airway stents [11].

Recent advances in additive manufacturing (AM) technologies (or 3D printing) are now revolutionizing the field of biomedical engineering, especially regenerative medicine, and tissue engineering. 3D printing has enabled the high-precision fabrication of patient-specific implantable devices with complex shapes that cannot be produced with conventional techniques. Today, airway stents are either constructed of silicone or metal, usually a nitinol, a nickel and titanium alloy. Hybrid stents are also available [12]. In addition, it is possible to 3D print biomaterials having mechanical properties compatible with the surrMatMODounding tissue, that can carry drugs or bioactive molecules, and have improved surface properties. The 3D printed airway stents need not have a fixed diameter, but self-expandable designs can also be utilized.

There are diverse AM methods, such as material extrusion, vat polymerization, powder bed fusion, material jetting, and sheet lamination techniques, each having specific advantages and disadvantages for different applications. In this study, a vat polymerization method, liquid crystal display (LCD)-based stereolithography (SLA) was preferred because of its high degree of accuracy, ability to produce parts with very high surface finish quality and relatively lower cost. This 3D printing technology is based on the use of any photosensitive resin, which polymerizes and hardens by ultraviolet (UV) rays with a specific wavelength. During 3D printing, an array of UV light is projected on a monochrome LCD panel which acts as a mask, revealing only the pixels necessary for selectively curing the photopolymer based on the image of each layer. When the resin in the vat is cured, the built platform rises out of the vat for the next layer as the part is built up in a layer-by-layer fashion.

3D printing can be applied in both direct and indirect ways. The main difference lies in the fact that the design can be directly produced from 3D printing (direct) or 3D printing can be used in the process of creating the model (indirect) with the use of 3D printed molds. The early attempts of rapid prototyping of customized airway stents were mostly based on indirect methods. For example, Chiang et al. [13] created a stent master pattern via SLA and then used it to make a silicone mold through the silicone vacuum casting process, and finally fabricated stents using these silicone molds. However, airway stent designs with open porous surfaces have been shown to be very difficult to remove from the mold [7]. Even in some cases, the process was reported to fail as the stent material -mostly silicone- had bonded with the molding material [14]. Therefore, direct 3D printing of porous stents instead of forming them on a mold is important both to benefit from the advantages of 3D printing technology and to ensure that complex geometries are produced in full compliance with the model. Among the various 3D design methods, function-based modeling is of great interest due to its high controllability for designing complicated pore architectures [15].

The current article aims to show how the mathematical surface functions can be used to design porous fixed-diameter semirigid airway stent structures with refined architectures for high-resolution stereolithographic 3D printing for the first time in literature and reveal the effects of 3D design in tailoring the mechanical properties and porosity of airway stents.

## Methods

### Materials

An opaque white, soybean-based, and biodegradable photopolymer resin (ECO UV Resin, Anycubic, China) was used for the fabrication of airway stents for demonstration purposes. The resin is sensitive to photocuring wavelengths of 355 nm-410 nm and compatible with liquid resin curing 3D printers with different technologies, such as stereolithography (SLA) and digital light processing (DLP). The properties of the resin were listed in Table 1 according to the information supplied by the manufacturer.

**Table 1:** The properties of the resin used in 3D printing [16]

Hardness	84D
Viscosity (at 25 °C)	150–300 MPa·s
Shrinkage	3.72–4.24%
Solid density	1.05–1.25 g/cm^3^
Bending strength	59–70 MPa
Extension strength	36–52 MPa
Thermal deformation	80 °C
Elongation at break	11–20%
Thermal expansion	95 × 10^–6^

### Stent design

**Fig. 1:**
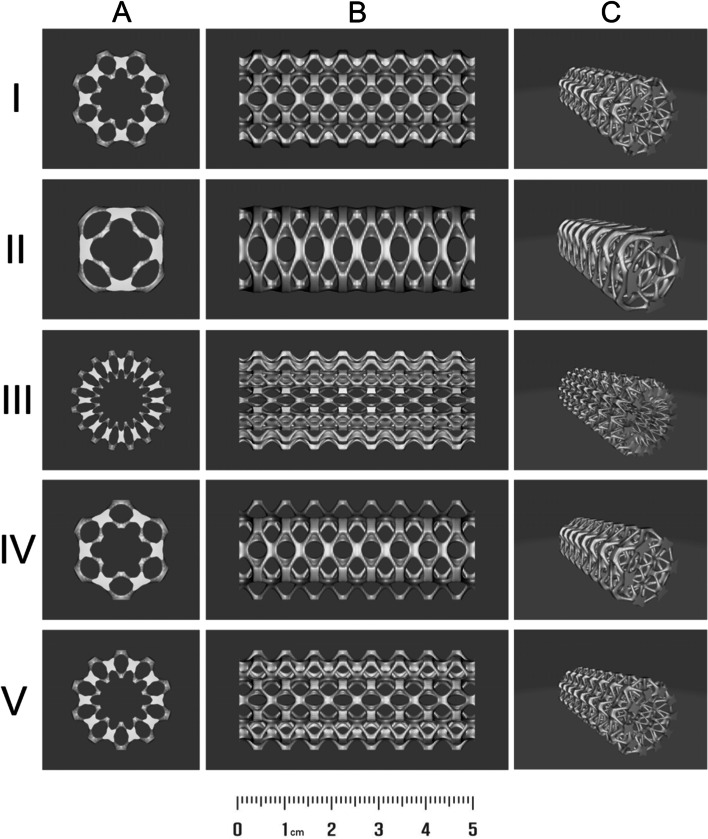
The renders from solid modeling of five different designs of airway stents (I-V). Three views: (**A**) top; (**B**) lateral; (**C**) oblique

The geometric architectures of the airway stents were generated by using an open-source mathematical modeling software MathMod version 10.1. The 3D mathematical surfaces were based on the ‘W_Skeletal Cylinder’ script based on the equation from the MathMod collection of mathematical drawing functions. Various 3D structures were obtained by changing the constant ‘N’, producing a set of 3D models that are the outputs of the same function. The N values were set to 8, 4, 16, 6, and 10 for the 3D models I, II, III, IV, and V, respectively. The renders from solid modeling of five different designs of airway stents are given in Fig. 1. The MathMod script of the mathematical function for the generation of the 3D models of the airway stent-I is given in the Supplementary Information.

### Stereolithographic 3D printing and post-processing

To convert the output of the MathMod (.obj files) into 3D printable format (.stl file) the 3D Builder modeling software (Microsoft Corporation), which automatically caps the polygonal mesh to obtain solid object, was used. 3D stereolithographic files were sliced using the Anycubic Photon Workshop software. The airway stents were manufactured by using a UV resin curing 3D printer (Anycubic Photon Mono, Shenzhen, China) by setting the outer diameter to 20 mm, since it is the closest size to the diameter of the trachea in humans [17]. The remains of uncured resin were washed away using dishwashing soap and distilled water instead of isopropyl alcohol (IPA) since the photopolymer resin is soybean-based and biodegradable. Finally, a laboratory-made UV post-curing machine (365 nm, 36 Watts) was used to ensure the complete curing of the end parts.

### Scanning Electron Microscopy (SEM)

The fine details in the microstructure of the 3D printed airway stents were characterized employing a desktop scanning electron microscope (SEM; JEOL, JCM-7000, Japan) in high vacuum mode. The airway stents were observed by a secondary electron detector by using 5 kV acceleration voltage. SEM analysis was carried out without gold coating in a sample chamber that allows direct examination of large samples.

### Physical properties

The density and volume of 3D printed airway stents were measured using a density determination kit of a laboratory balance (Ohaus, Switzerland) by following Archimedes’ principle.

Density $\left(\rho \right)$ of the airway stents were calculated according to following equation:

1 $$\uprho =\frac{A}{A-B}\left({\rho }_{0}-{\rho }_{L}\right)+{\rho }_{L}$$

and the volume $\left(V\right)$ of the airway stents was given by:

2 $$V=\alpha \frac{A-B}{{\rho }_{0}-{\rho }_{L}}$$

where $A$ is the weight of the sample in air, $B$ is the weight of the sample in water, ${\rho }_{0}$ is the density of water (0.9982 g/cm^3^ at T = 20ºC), ${\rho }_{L}$ is the density of air (0.0012 g/cm^3^), and $\alpha$ is the balance correction factor (0.99985) which takes air buoyancy of the adjustment weight into account [18].

Porosities of the 3D structures were calculated as follows:

3 $$Porosity (\%)=\frac{Bounding Volume-Measured Solid Volume}{Bounding Volume}\times 100$$

Additionally, a computer aided design (CAD) platform (Rhino7) was used to obtain the bounding volume, 3D model volume, and 3D model surface area of the airway stents.

### Mechanical properties

The compression tests of airway stents were performed using a universal testing machine (3367, Instron Co., USA) with a 30kN load cell capacity at a rate of 2 mm min^−1^. Every sample was tested three times.

## Results

**Fig. 2:**
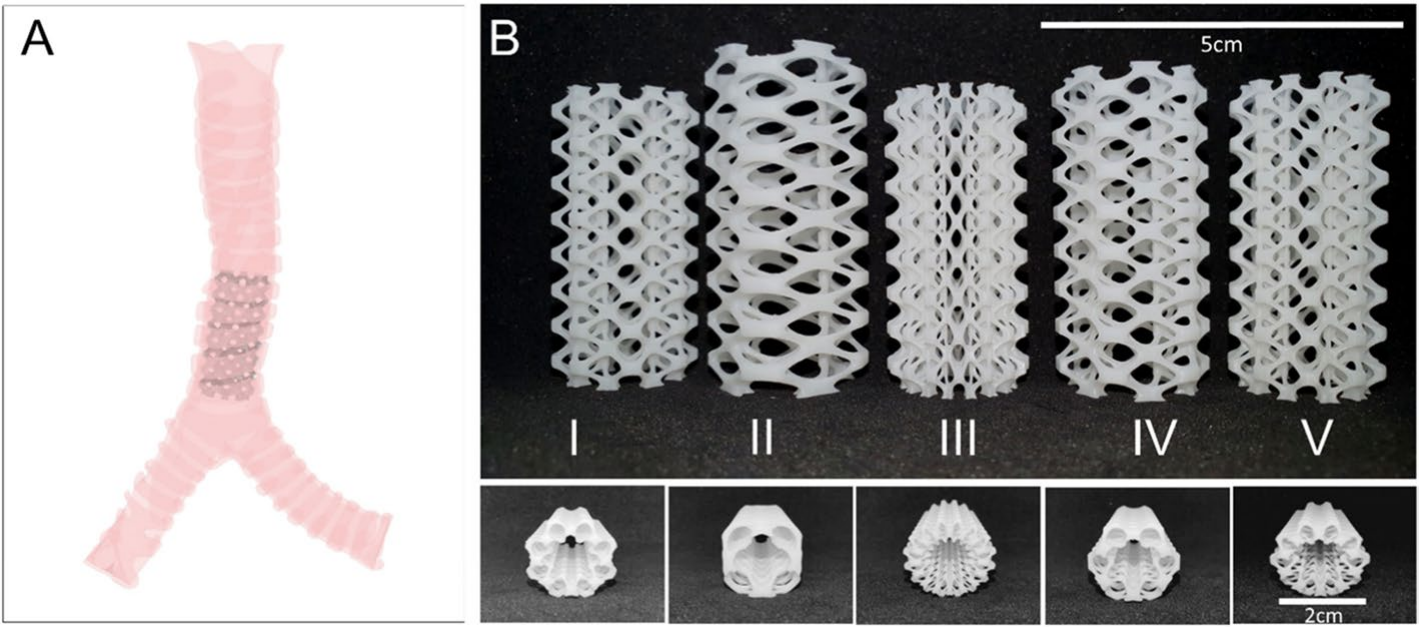
(**A**) Placement of an airway stent (porous tube) in the trachea. (**B**) The side and top views of the 3D printed airway stents (I-V) designed by using a mathematical surface function

Figure 2 shows the top and side view images of the 3D printed airway stents. Each group exhibits a unique pore geometry defined by their mathematical surface function-based designs. These macroscopic images showed that by using the LCD-based SLA 3D printer, airway stents were printed neatly and well in various complex patterns with well-defined geometry and interconnected channels.

**Fig. 3:**
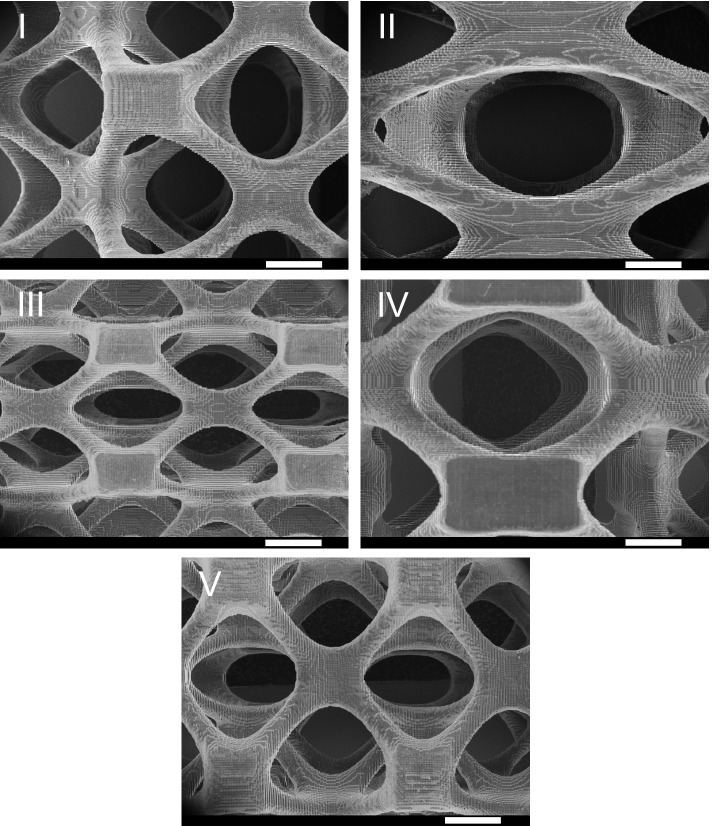
SEM micrographs showing the surface morphology of 3D printed airway stents (I-V) (Scale bars are 2 mm)

SEM micrographs of 3D printed airway stents (Fig. 3) showed the very finely detailed surfaces of 3D printed airway stents that are cured according to the pixels in each layer. The surface texture is determined mainly by the feature resolution of the 3D printer in the horizontal and vertical directions. The 3D printer used in this study has a 2 K monochrome LCD screen with a resolution of 2156 × 1621 giving a pixel size of 51 μm [19]. The Z-axis resolution of the printer is 10 μm and the 3D printing layer height was selected as 50 μm. The size of the cubic block-like structures on the surfaces of airway stents is ~ 50 μm, as revealed by SEM images. The pore sizes differ between groups depending on the design. The largest elliptical-shaped pore belongs to the airway stent II with a major axis of 10 mm.

Table 2 summarizes the physical properties of the airway stents. The measured porosity values were determined in very close agreement with the porosities of the CAD models. The mean porosity of all groups according to CAD models was determined as 87.42 ± 1.57. In addition, the mean density of the UV cured airway stent material was measured as 1.21 g/cm^3^.

**Table 2:** Physical properties of the airway stents

Model No	I	II	III	IV	V
Diameter (mm)	20	20	20	20	20
Height (mm)	48	54	47	50	47
Bounding Volume (mm^3^)	15.114	16.974	14.850	15.598	14.885
3D Model Volume (mm^3^)	1819	2587	1691	1980	1718
3D Model Surface Area (mm^2^)	6102	6636	7868	6007	6373
Measured Density (ρ) (g/cm^3^)	1.20	1.20	1.21	1.21	1.22
Measured Volume (V) (mm^3^)	1946	2659	1775	2020	1774
Porosity (%) acc. to Measured Volume	87.13	84.33	88.05	87.05	88.08
Porosity (%) acc. to 3D Model Volume	87.96	84.76	88.61	87.30	88.46

**Fig. 4:**
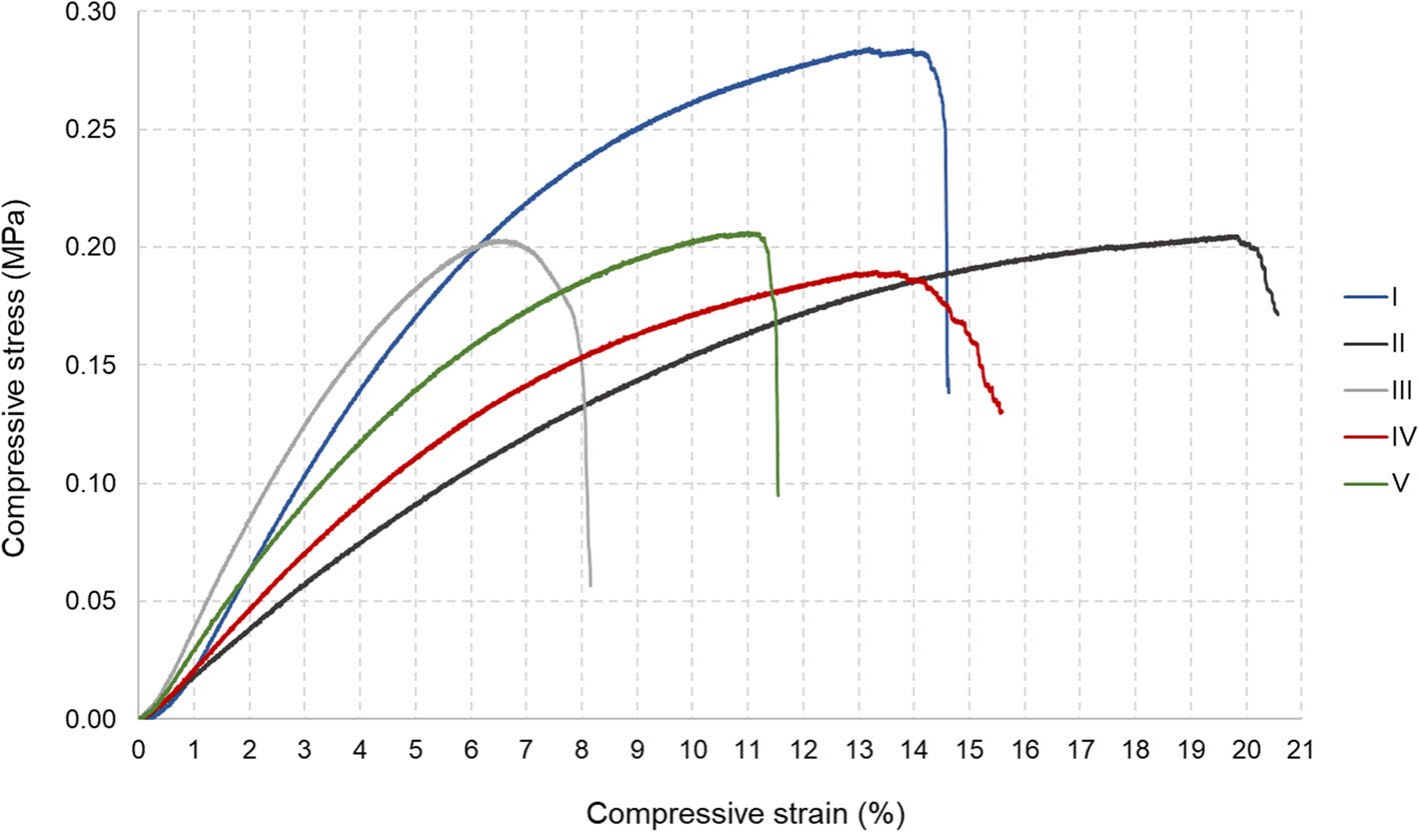
The compressive stress–strain curves of 3D printed airway stents (I-V)

Compression test was applied to each sample to determine whether the 3D design influence the mechanical strength of airway stents. The representative stress–strain curves of the five different groups are given in Fig. 4.

The maximum compressive strength along the longitudinal direction of the airway stents II, III, and V was measured as 0.205, 0.203, and 0.206 MPa, respectively, which were very close to each other. While airway stent IV exhibited a compressive strength of 0.190 MPa, airway stent I had a considerably higher strength compared to the other stents (0.284 MPa). Compressive strain is the change in length per original length due to the compressive force on the object. It was observed that the compressive strain at the yield point of all the stents changes between 6.5–20%. The modulus of elasticity of the airway stents I, II, III, IV, and V in compression was determined as 4.11, 1.96, 4.55, 2.52, and 3.43 MPa, respectively.

## Discussion

Currently available airway stents still have many issues that remain unresolved, such as the development of granulation tissue or mucus plug in the lumen. In addition, airway stents can easily become dislodged (stent migration) if they don’t fit properly. The failure rate for all stenting procedures is currently reported as high as 22%. With the increasing prevalence of lung cancer globally, the need to update airway stent technology is more important today than ever before and customizing stents using 3D printing technology may offer the best chance to address many of the current limitations in stent design [20].

Although the currently available silicone stents can be selected to fit the airway anatomy of a patient and have a defined, fixed-diameter that prevents uncontrolled expansion [21], they have the disadvantage of relatively small internal diameters but large external diameters. The main goals for the design of the fixed-diameter stents produced in this study were to obtain open pores to allow mucus drainage and to resist migration in the airway via the outer ridges. In addition, to provide a mechanically durable structure, the designs consist of a network of interconnected struts and well-arranged nodes in a 3D space. These sophisticated airway stent geometries cannot be achieved by using conventional 3D unit cells, such as cubic or truncated cellular structures. The math-based modeling approach offers significant advantages in enabling the generation of different pore geometries and architectural features [22]. The applicability of this approach has been demonstrated before, especially in areas such as bone tissue engineering. Tripathi et al. [23] demonstrated the generation of mathematical equation-based triply periodic minimal surfaces (TPMS) gyroid scaffold geometry with precisely controlled spatial porosity through interconnected pores and 3D printing of these structures. Furthermore, since traditional techniques like injection molding have design limitations, 3D printing offers revolutionary benefits in the production of such objects. The versatility of stereolithography in terms of materials and freedom of design has also been demonstrated in fabricating mathematically defined tissue-engineered scaffold architectures [24]. Therefore, the outstanding advantages of both design and production methods were synergically utilized in this study. The results of the macroscopic and microscopic evaluation of 3D printed airway stents showed the highly porous well-defined architectures of all groups. It was also shown how the mechanical strength can be tailored only by design considerations while keeping the material and manufacturing method constant in the manufacture of 3D printed airway stents even with very close porosity values. For example, by changing N, which is the constant in a mathematical function used in the stent design, caused the elastic modulus to differ. The ultimate compressive strength of the stent models II, III and V were very close while the compression strains are quite different from each other. Many factors may affect the stress and strain behavior under a constant load, such as the pore geometry, pore orientation, the number of struts, the strut size and the nature of the strut nodes of the samples when the production material is the same. To reveal the multiple relationships between these factors and mechanical behavior, it may be useful to perform finite element analysis instead of performing many independent mechanical tests. Using finite element analysis can reduce the number of physical prototypes and experiments performed while optimizing all components during the design phase.

Hoffman and colleagues performed a confined compression test to native porcine trachea and reported that the circumferential elastic modulus was 5.6 ± 2.0 MPa and longitudinal composite elastic modulus was 1.1 ± 0.7 MPa [25]. In addition, as previous studies reported the human tracheal modulus in the range 1–20 MPa [26], the airway stents with modulus ranging from 1.96–4.55 MPa produced in this study will not exhibit mechanical properties incompatible with the native tissue.

Advances envisioned for future airway stenting technologies focus on drug delivery as well as personalized stents. The drug-eluting airway stents can carry many agents, such as chemotherapeutics, radioactive substances, corticosteroids, sirolimus, mitomycin C and antibiotics [27]. For example, paclitaxel-loaded liquid silicone rubber containing polydimethylsiloxane stents were fabricated via injection molding [28] for the localized delivery of paclitaxel and minimized side effects. Our study opens the door to the possibility that any mechanically suitable drug carrying resin biomaterial that can be polymerized at a wavelength around 405 nm can be used for the manufacturing of airway stents since SLA is a low temperature 3D printing technique that does not lead to thermal degradation of drugs.

Current practices on 3D printing in airway stenting are very limited and focused mainly on providing the base data for reconstructing the airway of a patient from the axial CT scan images for simulation purposes [29]. On the other hand, one of the strongest aspects of 3D printing technologies is the direct manufacturing of the implants or scaffolds from digital 3D models converted from a DICOM (Digital Imaging and Communications in Medicine) file generated by using medical imaging of a patient. For example, Shan et al. [30] customized airway stents with the aid of 3D printing the models based on computerized tomography (CT) scans. The stent was reported to be technically successful in all 12 patients, and 11 of the patients (91.7%) and showed significant palliation of dyspnea after stenting. Fiorentino et al. [31] also converted 2D DICOM images acquired by CT or magnetic resonance (MR) imaging to a 3D model of the trachea which was used for deriving tracheal stents with smooth, spiral, and dome geometries. These studies, therefore, demonstrate that the implantation of novel stents manufactured with the aid of 3D printing is feasible for treating malignant or benign strictures of the tracheobronchial tree. The use of 3D-engineered, patient-specific stents should theoretically significantly reduce the risk of migration, granulation tissue reaction, and mucus occlusion [32]. Despite the significant advances in research on 3D printing of biomaterials, it is still a major shortcoming that the full potential of 3D printing for patient-specific implants and tissue reconstruction is not being exploited in the clinic. It has been suggested that the main reason for this is the lack of integration of image-based patient-specific design with 3D biomaterial printing within the scope of a regulatory framework, namely design control, required by the FDA [33].

## Conclusions

An LCD SLA 3D printer was employed for the fabrication of airway stents with complex architecture that were designed by using a mathematical surface function for the first time. The diversity of the designs was achieved simply by changing a mathematical constant. After using a series of software tools, the mathematical function-based designs were converted into 3D printable models and the resulting airway stents exhibited a very high porosity which can help prevent mucus accumulation. In addition, it has been shown that compressive strength and strain can be tailored by only creating a difference in 3D model design without changing the material and production method. Since the method used in this study applies to all materials that can be UV-cured at a certain wavelength, it is also suitable for 3D printing of drug-loaded biodegradable biomaterials with improved biocompatibility and mechanical properties. The process applied in this study also easily supports production according to patient-specific measurements based on medical imaging data.

## Supplementary Information

**Additional file 1.** Script from MathMod library to generate 3D airway stent model - I.

## Data Availability

All data generated or analysed during this study are included in this published article and its supplementary information file.
