# Supplementary material for: Mathematical surface function-based design and 3D printing of airway stents
Source: 3D Print Med. 2022 Aug 6;8:24. doi: 10.1186/s41205-022-00154-8 (PMC9356489; doi:10.1186/s41205-022-00154-8)
Supplement: Supplementary file 1 — Additional file 1. Script from MathMod library to generate 3D airway stent model - I. [file 41205_2022_154_MOESM1_ESM.docx]

**Supplementary Information**

***Script from MathMod library to generate 3D model of airway stent - I***

{

"Iso3D": {

"Component": [

"W_SkeletalCylinder"

],

"Const": [

"M=1",

"N=8",

"P=4"

],

"Description": [

"W_Skeletal Cylinder by Abderrahman Taha 01/11/2019"

],

"Funct": [

"W_Skeletal=cos(2*x)+cos(2*y)+cos(2*z)-2*(cos(x)*cos(y)+cos(y)*cos(z)+cos(z)*cos(x))+25/10",

"isoCondition_0=(x^2-(pi+1/2)^2)",

"isoCondition_1=(x^2-(pi+3/10)^2)",

"isoTransform=if(isoCondition_0(x,y,z,t)<(0),W_Skeletal(x,y,z,t),1)+M*exp(isoCondition_1(x,y,z,t))",

"isoTransform2=isoTransform((sqrt(x*x+z*z)-8),y,N*atan2(z,x),t)"

],

"Fxyz": [

"-isoTransform2(x,y,z,t)"

],

"Name": [

"W_Skeletal_Cylinder"

],

"Xmax": [

"11"

],

"Xmin": [

"-11"

],

"Ymax": [

"26"

],

"Ymin": [

"-26"

],

"Zmax": [

"11"

],

"Zmin": [

"-11"

]

}

}
